# Local structure and oxide-ion conduction mechanism in apatite-type lanthanum silicates

**DOI:** 10.1080/14686996.2017.1362939

**Published:** 2017-09-04

**Authors:** Olivier Masson, Abid Berghout, Emilie Béchade, Jenny Jouin, Philippe Thomas, Toru Asaka, Koichiro Fukuda

**Affiliations:** ^a^ Science des Procédés Céramiques et de Traitements de Surface (SPCTS), CNRS, Centre Européen de la Céramique, Limoges Cedex, France; ^b^ Department of Environmental and Materials Engineering, Nagoya Institute of Technology, Nagoya, Japan

**Keywords:** Lanthanum silicate, local structure, conduction mechanism, pair distribution function (PDF), density functional theory (DFT), 50 Energy Materials, 107 Glass and ceramic materials, 207 Fuel cells / Batteries / Super capacitors, 401 1st principle calculations, 504 X-ray / Neutron diffraction and scattering

## Abstract

The local structure of apatite-type lanthanum silicates of general formula La_9.33+x_(SiO_4_)_6_O_2+3x/2_ has been investigated by combining the atomic pair distribution function (PDF) method, conventional X-ray and neutron powder diffraction (NPD) data and density functional theory (DFT) calculations. DFT was used to build structure models with stable positions of excess oxide ions within the conduction channel. Two stable interstitial positions were obtained in accordance with literature, the first one located at the very periphery of the conduction channel, neighbouring the SiO_4_ tetrahedral units, and the second one closer to the channel axis. The corresponding PDFs and average structures were then calculated and tested against experimental PDFs obtained by X-ray total scattering and NPD Rietveld refinements results gathered from literature. It was shown that of the two stable interstitial positions obtained with DFT only the second one located within the channel is consistent with experimental data. This result consolidates one of the two main conduction mechanisms along the c-axis reported in the literature, namely the one involving cooperative movement of O4 and Oi ions.

## Graphical abstract

**Figure:**
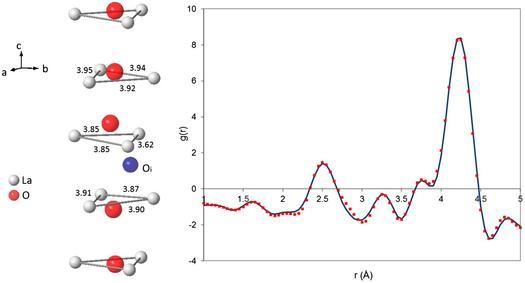

## 1. Introduction

**Figure 1.**
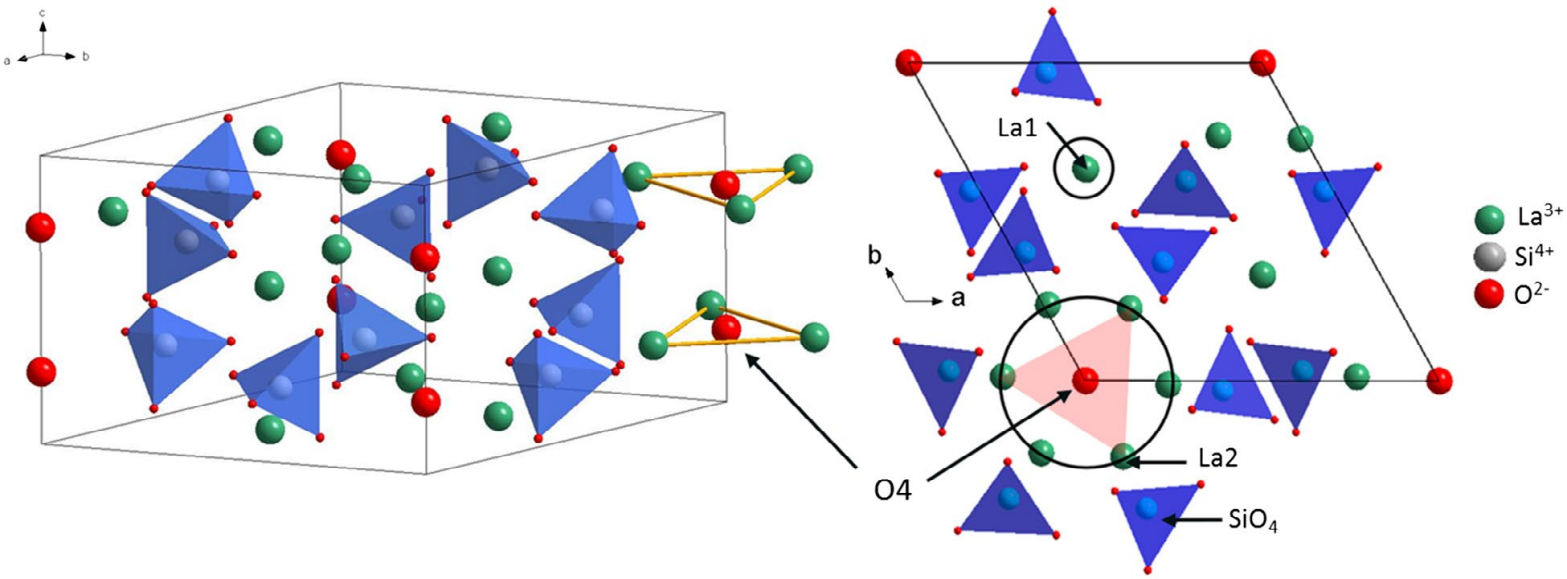
Unit cell of the reference apatite-type lanthanum silicate structure viewed in perspective (left image) and in projection along the c-axis**.**

Apatite-type lanthanum silicates of general formula La_9.33+x_(SiO_4_)_6_O_2+3x/2_ (0 < x < 0.27) are promising materials for gas-sensing devices and solid oxide fuel cell (SOFC) electrolytes because of their high oxide-ion conductivities at moderate temperatures as well as at low oxygen partial pressures [1,2]. Their conductivities are strongly anisotropic, more than one order of magnitude larger along the crystalline c-axis than perpendicular to it, in the temperature range from 623 K to 848 K [3]. They are also very sensitive to the amount of extra oxide ions present in the structure [4–6]. The conductivities of the La_9.50_(SiO_4_)_6_O_2.25_ compound measured at 723−973 K are, for example, larger by a factor of 2.5 with respect to those of the oxygen stoichiometric La_9.33_(SiO_4_)_6_O_2_ compound [6]. Their structure is made of isolated SiO_4_ tetrahedral units forming two distinct channels parallel to the c-axis (see Figure 1) [7,8]. The smaller of these channels contains La1 lanthanum cations and vacancies, while the larger one, called the conduction channel, contains both La2 lanthanum cations and O4 oxide ions. The O4 ions do not belong to tetrahedral units and instead are located on the channel axis, at the centre of equatorial La2 triangles. The remaining oxide ions (O1–O3) are bonded to Si cations, forming SiO_4_ units. The O3 ions border the conduction channels, O1 are a little further away and O2 are the furthest ones.

Since the first reports of Nakayama et al. [1,2], there have been a number of experimental and computational studies devoted to the understanding of these materials, in particular their conduction mechanism along the c-axis [9–15]. Most results support that conduction along the c-axis involves an interstitial mechanism with interstitial sites located in the conduction channel [9–26]. However, precise answers regarding the exact interstitial mechanism responsible for the high ionic conductivity and the related interstitial sites for excess oxide ions are still unclear, though essential for further improvement of the performance of these materials. Two main mechanisms have been proposed in the literature so far. In a pioneering work, Tolchard et al. [9,10] and Jones et al. [11] proposed, using atomistic simulations, a direct interstitial mechanism involving a ‘sinusoidal-like’ migration pathway connecting interstitial sites Oi located at the periphery of the conduction channel. They showed that the incorporation of interstitial oxide ions should lead to the formation of distorted SiO_5_ units bordering the channel. In their mechanism, the migration energy was found to be 0.56 eV, and O4 atoms did not play a direct role. Shortly afterwards, some of the present authors [12] proposed, again using atomistic simulations, a non-collinear interstitialcy mechanism in which interstitial ions cooperatively move with O4 ions along a nonlinear pathway. The calculated migration energy of this mechanism was 0.32 eV, and the interstitial sites Oi were found to be located within the conduction channel, closer to the channel axis. Since then, various studies have argued in favour of one or the other of the mechanisms. For example, recent measurements of the activation energies of the conduction along the c-axis for the La_9.33_(SiO_4_)_6_O_2_ and La_9.50_(SiO_4_)_6_O_2.25_ compounds [27] gave values of 0.35 eV, in good agreement with that calculated for the interstitialcy mechanism. Similarly, some first-principles calculations [13–15] and one neutron powder diffraction (NPD) experiment [26] gave results consistent with the interstitialcy mechanism and its associated interstitial position. On the other hand, a number of experimental structural studies, mainly based on diffraction and NMR experiments, suggested that the interstitial sites were located at the periphery of the conduction channels [18–23]. This is notably the case for a series of NPD studies [18–20,22] performed on various oxygen excess and oxygen-stoichiometric compounds from low (4 K) to higher temperatures (1173 K). In these latter studies, the large atomic displacement parameter (ADP) values obtained for the O3 ions were considered as indicative of the structural disorder induced by channel periphery interstitial ions and were found consistent with fit improvement and Fourier difference map analyses.

The exact positions of the interstitial oxide ions, as well as the related structural distortions, are thus not known yet. And although computational methods are very useful in this respect, experimental evidences remain crucial even if they are often subtle and not univocal. The aim of the present paper is to reinvestigate the location of the interstitial oxide ions by combining the atomic pair distribution function (PDF) method, conventional X-ray and neutron diffraction data and density functional theory (DFT) calculations. DFT is used to build structure models with stable positions of the extra oxide ions in the conduction channel. The corresponding PDFs and average structures are then calculated and tested against experimental PDF obtained by X-ray total scattering and NPD Rietveld refinement results gathered from the literature. Given the relative closeness of the atoms in the conduction channel, the insertion of extra oxide ions should induce detectable local distortions of the structure, affecting not only the closest oxide ions but also lanthanum cations forming the triangles. This should affect the average structure in terms of atomic positions and ADPs, which are assessed by conventional diffraction methods, and also the PDF obtained by X-ray total scattering, which should be sensitive to the local distortions in the surrounding lanthanum sub-lattice.

## 2. Experimental details

### 2.1. Synthesis

Two samples of nominal compositions La_9.33_(SiO_4_)_6_O_2_ and La_9.50_(SiO_4_)_6_O_2.25_ were prepared by conventional solid-state reaction from high-purity La_2_O_3_ (Alfa Aesar, Karlsruhe, Germany; crystalline, 99.99%) and SiO_2_ (Prolabo, Coueron, France; amorphous, 99.9%) powders. Small quantities of the starting powders (a total of ~0.2 g) were intimately mixed in the correct ratios using an agate mortar and pestle and then placed in Pt sealed tubes. The whole procedure was performed in a nitrogen dry glove box (MBRAUN, Garching, Germany; O_2_ < 0.1 ppm and H_2_O < 0.1 ppm) to avoid the hydration of La_2_O_3_. The mixtures were heated first to 1300 °C for 14 h, reground, and then reheated to 1400 °C for a further 14 h to achieve a complete reaction. Phase purity was checked through X-ray powder diffraction (Bruker D8 advance diffractometer with CuKα1 radiation and rapid LynxEye detector; Wissembourg, France). No impurities, in particular La_2_SiO_5_, were detected in either sample (Figure S1, Supporting information).

### 2.2. Average structure and PDF measurements

The average structures were determined using conventional diffraction data measured on a Bruker D8 advance diffractometer with CuKα1 radiation and rapid LynxEye detector. Data acquisitions were performed from 17° to 148° (2θ) with a step size of about 0.01°. Long acquisition time of 72 h was used in order to obtain good counting statistics data. The resulting diffraction data were analysed by the Rietveld method [28] using the program FullProf [29] (the complete program and documentation can be obtained at http://www.ill.eu/sites/fullprof/). Atomic positions and anisotropic displacement parameters were refined in space group P6_3_/m. No excess oxide ion was explicitly considered. Occupation factors at the La1 site were refined in order to check the sample compositions.

The PDF of the samples were obtained via X-ray total scattering data measured with a home-made two-axis diffractometer (MoKα radiation, 0.71069 Å, and scintillation counter). For each sample, a few milligrams of powder were placed in a thin-walled (0.01 mm) borosilicate glass capillary of 0.3 mm diameter. Measurements were performed at room temperature from Q = 0 to Q = 17 Å^−1^ (Q = 4πsinθ/λ) with a step size of 0.02 Å^−1^, an acquisition time of 960 s per step and sample rotation about its axis. Additional measurements of the scattering from empty capillary and empty environment were performed in the same conditions for subsequent data corrections. Raw data were treated using a home-made program written in python language. Data were first corrected for capillary and air scattering, absorption, polarization, Compton scattering, fluorescence and Kα_2_ radiation. They were then normalized and reduced into the structure function and finally Fourier-transformed into the total PDF.

## 3. Computational details

### 3.1. Model construction

Three structure models were constructed, namely the oxygen stoichiometric La_9.33_(SiO_4_)_6_O_2_ model without interstitial oxide-ion (denoted as M1 hereafter) and two different oxygen excess La_9.50_(SiO_4_)_6_O_2.25_ models, one with Oi within the channel [12] (denoted as M2) and the other with Oi at the channel periphery [9] and forming SiO_5_ unit (M3). Lanthanum vacancies and extra oxide ions were taken into account by creating supercells. A (1×1×3) supercell (i.e. three times the conventional hexagonal unit cell along the c-axis) containing 124 atoms and 2 lanthanum vacancies was used for the La_9.33_(SiO_4_)_6_O_2_ model. For the two La_9.50_(SiO_4_)_6_O_2.25_ models, a (1×1×4) supercell containing 167 atoms, including 1 extra oxide ion, and 2 vacancies was used. As suggested in experimental studies [8,18], the two La vacancies were placed at the La1 sites and far away from each other to avoid vacancy clustering. This latter point was furthermore supported by recent DFT calculations on the effects of La vacancy configuration in which interaction between vacancies was shown to be repulsive [13]. The as-obtained starting configurations were then relaxed in order to reach their equilibrium geometries. This was performed in the framework of periodic DFT using the Vienna Ab initio Simulation Package (VASP; Vienna, Austria) [30,31]. VASP is a plane wave basis set code in which the Kohn–Sham equations are solved variationally. It attempts to match the accuracy of the most advanced all-electron codes by using a projector-augmented wave (PAW) approach [32,33] to describe the electron–ion interaction. For the exchange-correlation functional the generalized gradient approximation (GGA) was used in the formulation of Perdew and Wang (PW91) [34,35]. The valence state was treated using 5p, 6s and 5d (no f) orbitals for La, 3s and 3p orbitals for Si and 2s and 2p orbitals for O. Electronic wave functions were expanded up to a cutoff energy of 500 eV, and Brillouin zone integration was performed only at the gamma point to get a good accuracy of less than 1.4 meV per atom. Structure relaxations were carried out by minimizing the Hellmann–Feynman forces to less than 0.02 eV Å^−1^ using a quasi-Newton method [36], and constraining the metric of the supercells to the hexagonal symmetry (a = b, a = b = 90°, γ = 120°).

**Figure 2.**
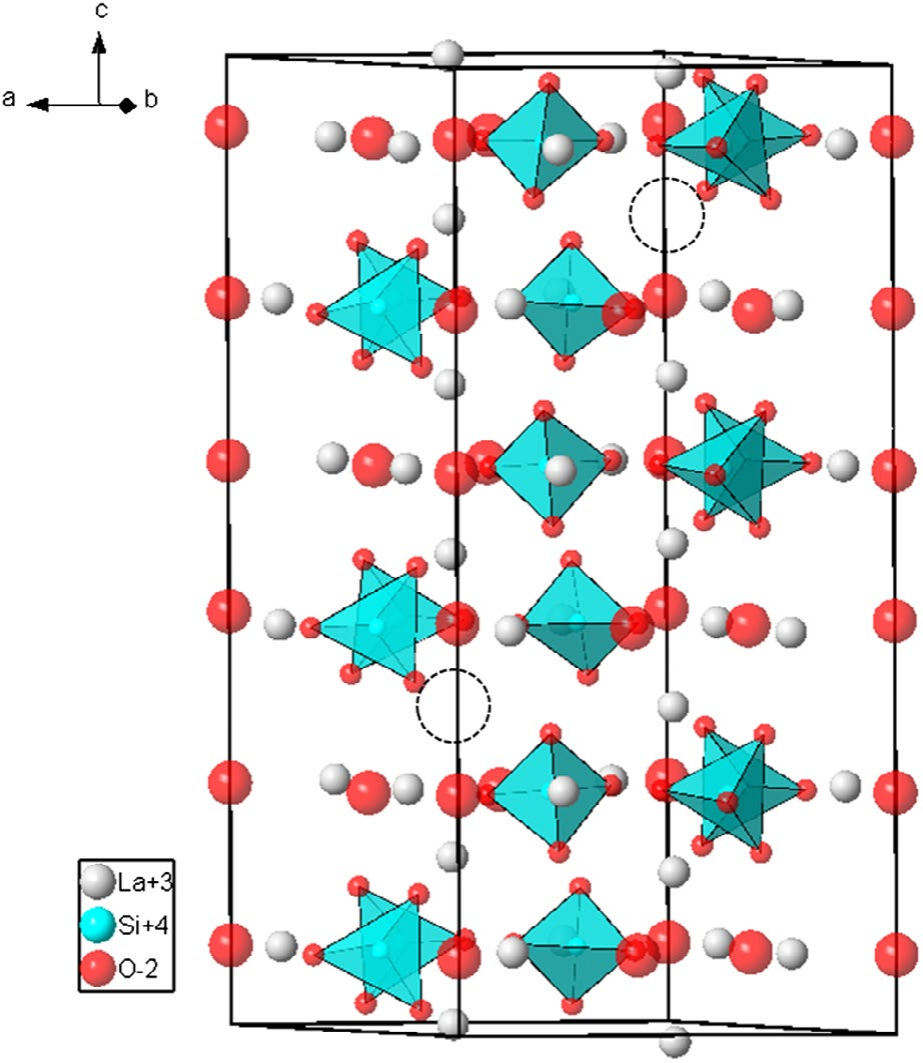
DFT-relaxed (1×1×3) supercell of the La_9.33_(SiO_4_)_6_O_2_ model (M1) used in this study. The empty broken-line circles indicate positions of La1 vacancies**.**

Stable configurations were obtained for the three models. The final supercell obtained for the La_9.33_(SiO_4_)_6_O_2_ model is plotted in Figure 2 as an example. The optimized lattice parameters (given in the conventional hexagonal unit cell) were a = 9.813 Å and c = 7.269 Å for the (1×1×3) supercell and a = 9.829 Å and c = 7.286 Å for the (1×1×4) supercells. These values agree with experimental data to within 1% error. For the model M2, the relaxed position of Oi was found within the channel at coordinates (–0.001, 0.091, 0.541), at 0.37 Å from previously reported values [12] and at 2.64 Å and 2.79 Å from the two neighbouring O4 ions. The distances between Oi and the three closest O3 ions are 2.69 Å, 2.78 Å and 3.03 Å. In the model M3, Oi was found at the channel periphery at coordinates (0.016, 0.223, 0.630), at 0.40 Å from previously reported value [18] and at 2.66 Å and 3.42 Å from the two neighbouring O4 ions. The distances between Oi and the closest O3 ions (belonging to the SiO_5_ unit) are 2.31 Å and 2.43 Å. The two next Oi-O3 distances are 2.78 Å and 3.13 Å. The computed energies of the two (1×1×4) supercells were equal to –1392.691237 eV and –1392.603047 eV for models M2 and M3, respectively. The energy of formation of interstitial oxide ions within the channel is thus lower by 0.088 eV, which favours its occurrence with respect to the SiO_5_ unit at 0 K. However, the energy difference is small enough to expect that SiO_5_ units may form at higher temperature.

### 3.2. Computation of the PDF and ADPs from the structure models

The three models described in the previous section were used to calculate the corresponding PDFs and ADPs of the asymmetric unit of the average unit cell. The PDFs were computed in the following way. First, the supercell dimensions of the models were adjusted to the experimental values. However, because the calculated and experimental c/a ratios were slightly different only the a-parameter was exactly adjusted to the experimental value (9.72 Å), and c was changed homogeneously to a slightly larger value (7.21 Å). This avoids introducing inhomogeneous distortions in the local structure. Second, the six partial PDFs (corresponding to La-O, Si-O, O-O, La-La and La-Si pairs) were calculated from the models by using the PDFFIT program [37] considering periodic boundary conditions. A small isotropic displacement parameter for each type of atoms (U_ii_ = 0.005 Å^−1^ for La, U_ii_ = 0.002 Å^−1^ for Si and U_ii_ = 0.003 Å^−1^ for O) was also considered in the calculation in order to broaden the PDF peaks in accordance with experiment. This mimics random atomic displacements due to thermal agitation, but not the (anisotropic) static disorder, the latter being contained in the supercells. Third, the partial PDFs were combined in order to obtain the total PDF. In contrast to the case of neutron scattering, the X-ray PDF is not a simple weighted linear combination of partial PDFs in particular for compounds with both heavy and light elements as lanthanum silicates. We treated this problem exactly, using the recent work of two of the present authors [38]. Finally, the effect of two major instrument aberrations (i.e. finite angular resolution and axial divergence of the scattered X-ray beams) was taken into account. Note that given the supercell dimensions and the use of periodic boundary conditions, the as-computed PDFs are not biased by minimum image distances only for correlation lengths lower than about 5 Å (i.e. half the smallest supercell dimension). This is largely enough for comparison to experimental data and to highlight local structure modifications.

The anisotropic ADPs of the seven atoms (La1, La2, Si, O1, O2, O3 and O4) of the asymmetric unit of the average unit cells were calculated in a more straightforward way. First, the positions of the atoms in the supercell were reduced to positions into a single unit cell (i.e. each supercell was projected onto a single unit cell). Second, the asymmetric unit was built using the 12 symmetry operations of the P6_3_/m space group. Third, the average positions and mean-square displacements were computed (excluding the out-of-site Oi atom) for the seven atoms of the asymmetric unit. Last, the as-calculated ADPs were multiplied by a global scaling factor of 1.5 for comparison with experimental values. The algorithm was coded in python language.

## 4. Results and discussion

### 4.1. Average structures analysis

**Figure 3.**
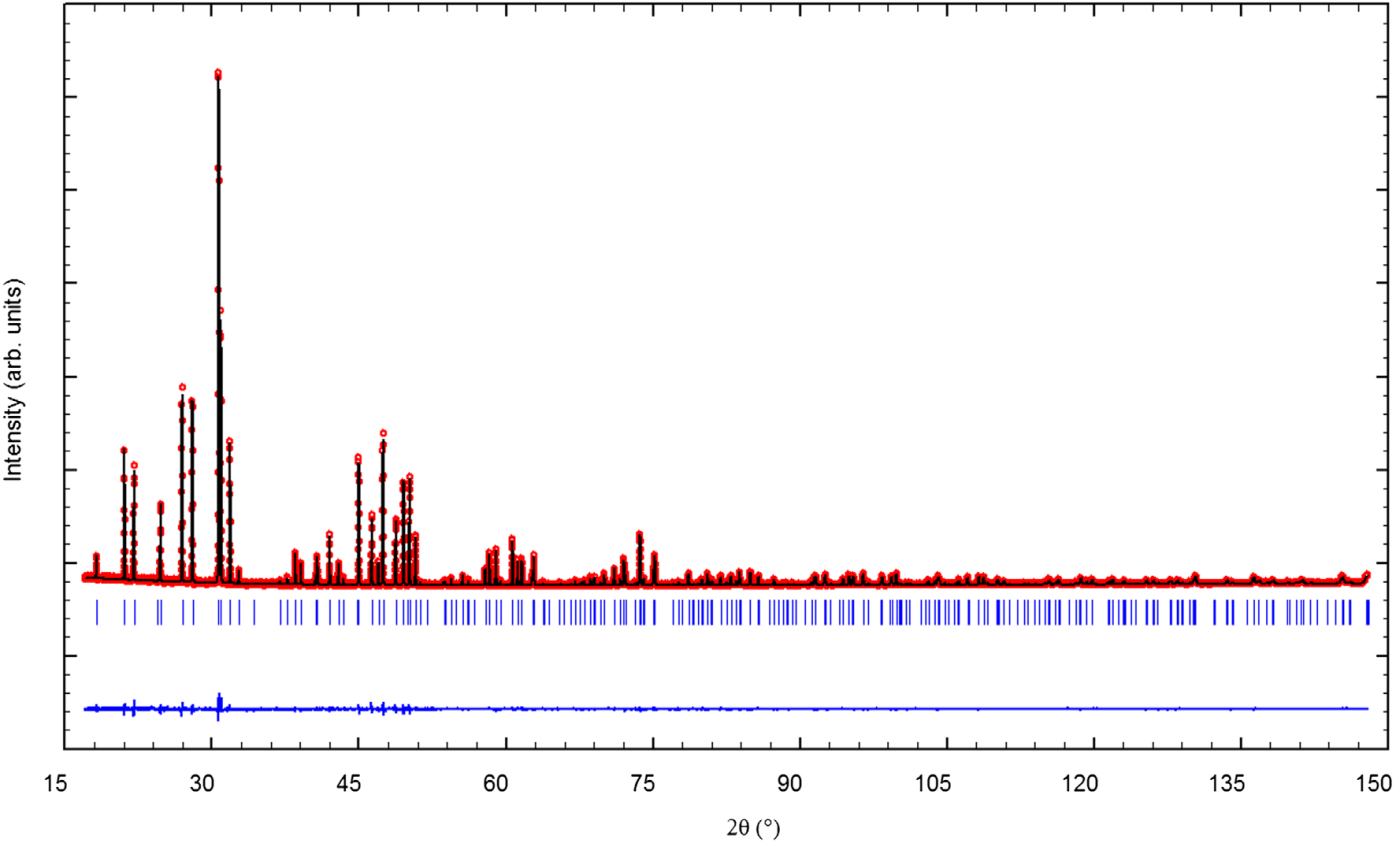
Rietveld fit for the L2 sample: observed (open circle), calculated (solid line) and difference patterns**.**

The refined structural parameters of the two samples are gathered in Tables 1, 2 and 3. The Rietveld fit is plotted in Figure 3 for the L2 sample only (see Figure S2, Supporting information, for the L1 sample fit). The La1 site occupancy values (Table 1) were refined to 0.847(1) and 0.872(1) for the samples L1 and L2, respectively. The corresponding chemical formulae are La_9.39_(SiO_4_)_6_O_2.08_ and La_9.49_(SiO_4_)_6_O_2.23_. These values are reasonably close to the nominal compositions and confirm that L2 contains more oxide ions in the conduction channel than L1. They also indicate that L1 is not perfectly oxygen stoichiometric as expected but exhibits some amount of excess oxygen. It can be observed from Table 1 that the cell parameters decrease from L1 to L2 with a larger decrease for the c-parameter, and the La2 triangle area clearly decreases with increasing oxygen excess content as previously reported in the literature [5].

**Table 1.** Reliability factors, cell parameters, La1 site occupancy, chemical formula and La2 triangle area for samples L1 and L2.

Sample	R_wp_ (%)	GofF	a	c	occ_La1_	Chemical Formula	S_La2_ (Å^2^)
L1	3.47	1.13	9.7244(1)	7.1871(1)	0.847(1)	La_9.39_(SiO_4_)_6_O_2.08_	6.88
L2	3.44	1.14	9.7232(1)	7.1839(1)	0.872(1)	La_9.49_(SiO_4_)_6_O_2.23_	6.78

**Table 2.** Refined atomic coordinates and ADPs (×10^−2^ Å^2^) for sample L1. * U_23_ of O3 was fixed during refinement.

Site	x	y	z	U_11_	U_22_	U_33_	U_12_	U_13_	U_23_	U_eq_
La1 (4f)	1/3	2/3	−0.0015(3)	0.71(5)	U_11_	2.23(5)	U_11_/2	0	0	1.22(5)
La2 (6 h)	0.0134(1)	0.2431(1)	1/4	0.36(4)	0.61(4)	0.65(2)	0.20(4)	0	0	0.56(3)
Si (6 h)	0.4009(5)	0.3721(4)	1/4	1.0(2)	0.6(2)	0.7(2)	0.4(1)	0	0	0.7(2)
O1 (6 h)	0.323(1)	0.480(1)	1/4	2.1(5)	3.7(7)	0.6(5)	1.8(5)	0	0	1.9(5)
O2 (6 h)	0.590(1)	0.471(1)	1/4	1.2(5)	0.4(3)	1.3(2)	0.3(4)	0	0	1.0(3)
O3 (12i)	0.3438(8)	0.2564(7)	0.0739(7)	5.9(5)	1.4(4)	0.8(3)	1.9(4)	−1.5(3)	−0.3*	2.6(3)
O4 (2a)	0	0	1/4	1.1(8)	U_11_	9(1)	U_11_/2	0	0	4(1)

**Table 3.** Refined atomic coordinates and ADPs (×10^−2^ Å^2^) for sample L2. * U_23_ of O3 was fixed during refinement.

Site	x	y	z	U_11_	U_22_	U_33_	U_12_	U_13_	U_23_	U_eq_
La1 (4f)	1/3	2/3	−0.0012(3)	0.71(5)	U_11_	1.97(5)	U_11_/2	0	0	1.13(5)
La2 (6 h)	0.0127(1)	0.2411(1)	1/4	0.41(4)	0.80(4)	0.69(2)	0.22(4)	0	0	0.67(3)
Si (6 h)	0.4027(5)	0.3727(4)	1/4	1.1(2)	0.6(2)	0.4(1)	0.5(1)	0	0	0.7(2)
O1 (6 h)	0.325(1)	0.484(1)	1/4	1.9(5)	4.2(7)	0.8(5)	2.6(5)	0	0	1.9(5)
O2 (6 h)	0.591(1)	0.471(1)	1/4	1.8(5)	0.7(4)	1.2(5)	0.6(4)	0	0	1.3(4)
O3 (12i)	0.3459(8)	0.2578(7)	0.0730(7)	6.2(5)	2.6(4)	1.6(4)	2.7(4)	−1.9(3)	−0.3*	3.3(4)
O4 (2a)	0	0	1/4	0.9(7)	U_11_	25(2)	U_11_/2	0	0	9(1)

The refined ADPs are reported in Tables 2 and 3. Relatively high values are observed for the La1, O1, O3 and O4 sites. The highest values for lanthanum atoms correspond to U_33_ of La1. It slightly decreases from L1 to L2 samples, emphasizing its connection with the presence of lanthanum vacancies at this site. As previously proposed in the literature [18,19,22], the main ADPs that are useful to detect local disorder in the conduction channel are U_11_ of O3 and U_33_ of O4. For both samples, U_11_ values of O3 are large and almost equal within experimental error. In contrast, a significant difference between the two samples is observed for U_33_ of O4, with about 9×10^−2^  Å^−2^ for sample L1 to a huge value of 25×10^−2^ Å^−2^ for L2.

In Table 4 are reported ADP values from the literature measured by NPD at room and at low (15 K) temperature [18,22] for the La_9.33_(SiO_4_)_6_O_2_ and La_9.55_(SiO_4_)_6_O_2.32_ compounds. Very similar ADP values measured by NPD at 3 K (not reported herein) were also obtained for the La_9.67_(SiO_4_)_6_O_2.5_ compound [26]. All these values are expected to be particularly precise for oxygen atoms and give valuable information relative to static and thermal disorder. As for samples L1 and L2, the highest values are observed for the La1, O1, O3 and O4 sites. For the La_9.33_(SiO_4_)_6_O_2_ sample, the small differences observed between the ADPs measured at room and low temperatures for the La1, O1 and O3 sites suggest that the corresponding positional disorder is mainly of static nature. For the O4 site, the difference between the room and low temperature U_33_ values is higher, indicating a larger contribution of thermal disorder. However, U_33_ of O4 at 15 K is relatively large (1.7×10^−2^ Å^2^) so that static disorder is not negligible. Regarding the La_9.55_(SiO_4_)_6_O_2.32_ sample, the main change concerns U_33_ of O4, which increases to a very high value of 21.1×10^−2^ Å^2^. Furthermore, the value of U_33_ of O4 is only slightly larger at 773 K [18], with a value of 22.6×10^−2^ Å^2^, suggesting that the corresponding disorder is again mainly of static nature.

**Table 4.** ADP values (×10^−2^ Å^2^) from the literature obtained by neutron diffraction at room temperature for La_9.33_(SiO_4_)_6_O_2_ [22] and La_9.55_(SiO_4_)_6_O_2.32_ [18]. Values in parentheses given for the highest ADP were obtained at 15 K [22].

	La_9.33_(SiO_4_)_6_O_2_ [22]						La_9.55_(SiO_4_)_6_O_2.32_ [18]					
*Site*	*U*_*11*_	*U*_*22*_	*U*_*33*_	*U*_*12*_	*U*_*13*_	*U*_*23*_	*U*_*11*_	*U*_*22*_	*U*_*33*_	*U*_*12*_	*U*_*13*_	*U*_*23*_
La1	0.3	U_11_	**2.4**(2.1)	U_11_/2	0	0	0.8	U_11_	**2.5**	U_11_/2	0	0
La2	0.6	0.2	0.3	0.1	0	0	1.0	0.5	0.5	0.2	0	0
Si	0.5	0.1	0.8	0.3	0	0	0.8	0.4	0.7	0.6	0	0
O1	**2.2**(2.9)	**2.3**(2.2)	**1.7**(0.8)	**2.0**(2.2)	0	0	**2.7**	**2.8**	**1.8**	**2.3**	0	0
O2	0.7	0.4	**2.0**(1.4)	0.2	0	0	0.9	0.7	**2.0**	0.4	0	0
O3	**4.5**(3.8)	**0.9**(0.9)	**1.1**(0.6)	**1.3**(1.1)	**−1.5**(−1.5)	**−0.6**(−0.5)	**4.8**	**1.5**	**1.3**	**1.7**	**−1.7**	**−0.8**
O4	0.3	U_11_	**6.7**(1.7)	U_11_/2	0	0	0.5	U_11_	**21.1**	U_11_/2	0	0

These values can now be compared to the ADPs calculated from the three models M1, M2 and M3 described in previous sections. All the calculated values are gathered in Table 5. It can be observed that about half of the calculated ADPs are close to zero and the other half are large. As already noticed, the calculated ADPs are indicative of static disorder only. Thus, small calculated values indicate that ADPs are mainly influenced by thermal motion of atoms, and large values (in bold in Tables 4 and 5), related to the La1, O1, O3 and O4 sites, indicate that ADPs are more influenced by static disorder in the structures. These latter values match well on average with experimental values. Regarding first the model M1, the calculated U_33_ of O4 (2.4×10^−2^ Å^2^) is consistent with the experimental value measured at 15 K (1.7×10^−2^ Å^2^). This confirms that static disorder is already present in the structure without an interstitial oxide ion. Indeed, O4 never exactly lies in the centre of the La2 triangle but is systematically shifted upward or downward along the c-axis (this is discernible in Figure 2). More interestingly, the calculated U_11_ value of O3 (3.6×10^−2^ Å^2^) is high and compares well with experiment. The associated static disorder, clearly seen on Figure 2, corresponds to large displacements of O3 ions along the a-axis induced by silicate tetrahedra tilts. Thus, in contrast to what was proposed in the literature [18,22], the large experimental value of U_11_ is not the signature of interstitial oxide ions at the periphery of the conduction channel but is mainly the signature of the intrinsic disorder in the oxygen stoichiometric structure. The same reasoning applies to the O1 site but to a lesser extent as this site is farther from the conduction channel than O3.

**Table 5.** ADP values (×10^−2^ Å^2^) calculated from the structure models M1, M2 and M3 presented in this paper.

	La_9.33_(SiO_4_)_6_O_2_ model M1						La_9.50_(SiO_4_)_6_O_2.25_ model M2 (with O_i_ within the channel)						La_9.50_(SiO_4_)_6_O_2.25_ model M3 (with O_i_ forming SiO_5_ unit)					
*Site*	*U*_*11*_	*U*_*22*_	*U*_*33*_	*U*_*12*_	*U*_*13*_	*U*_*23*_	*U*_*11*_	*U*_*22*_	*U*_*33*_	*U*_*12*_	*U*_*13*_	*U*_*23*_	*U*_*11*_	*U*_*22*_	*U*_*33*_	*U*_*12*_	*U*_*13*_	*U*_*23*_
La1	0.0	0.0	**3.5**	0.0	0	0	0.2	0.2	**2.4**	0.1	0	0	0.6	0.6	**2.5**	0.3	0	0
La2	0.0	0.0	0.2	0.0	0	0	0.3	0.2	0.9	−0.1	0	0	0.6	0.2	0.8	0.1	0	0
Si	0.0	0.1	0.1	0.0	0	0	0.2	0.3	0.2	0.2	0	0	0.2	0.2	0.6	0.1	0	0
O1	**1.0**	**1.6**	**1.7**	**1.1**	0	0	**1.4**	**1.8**	**2.3**	**1.4**	0	0	**1.5**	**1.9**	**2.7**	**1.5**	0	0
O2	0.1	0.3	**0.5**	−0.2	0	0	0.3	0.3	**1.4**	−0.1	0	0	0.3	0.4	**1.6**	−0.1	0	0
O3	**3.6**	**0.3**	**0.6**	**0.7**	**−1.4**	**−0.3**	**4.2**	**1.1**	**1.0**	**1.7**	**−1.8**	**−0.7**	**7.1**	**5.0**	**1.7**	**4.8**	**−3.2**	**−2.4**
O4	0.0	0.0	**2.4**	0.0	0	0	0.8	0.8	**22.4**	0.4	0	0	1.1	1.1	**3.4**	0.5	0	0

Comparing the two models M2 and M3 of the La_9.50_(SiO_4_)_6_O_2.25_ compound with respect to the model M1, major differences appear for the O3 and O4 sites. With model M2, the ADP values of O3 slightly increase and the U_33_ value of O4 rises dramatically to 22.4×10^−2^ Å^2^. The opposite behaviour is observed for the M3 model, namely a slight increase of U_33_ of O4 and a large increase of all O3 ADPs. Clearly, the M2 model is consistent with experiment, and its ADP values compare particularly well with the experimental ones. In contrast, the ADP values of the model M3 give the largest discrepancies with respect to experiment. The observed increase of U_33_ of O4 is thus the signature of interstitial oxide ions within the channel. Interstitial oxide ions at the periphery of the channel would induce no significant increase of U_33_ of O4 and a much larger increase of O3 ADP values than experimentally observed.

### 4.2. Atomic pair distribution functions analysis

**Figure 4.**
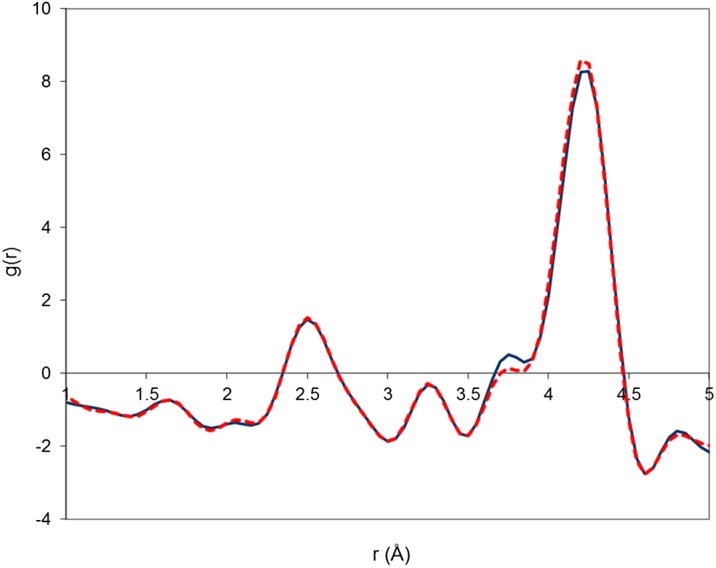
Experimental PDF of sample L1 (dashed line) and sample L2 (solid line) measured by X-ray total scattering**.**

The experimental PDFs of the two samples are plotted, in the range 1 < r < 5 Å, in Figure 4. They both exhibit features typical of the lanthanum silicate structure. The first peak at about 1.62 Å corresponds to Si-O bond lengths. The second peak, broader than the first one and centred at about 2.50 Å, corresponds to La-O bond lengths. The third peak at 3.25 Å corresponds to La-Si shortest distances. The peaks corresponding to O-O distances are not noticeable. The most intense peak at about 4.20 Å corresponds mainly to La-La distances with a small contribution of La-O distances. The shoulder at 3.80 Å is composed of La-La and La-Si distances. As expected, the differences between the two PDFs are small and related to correlation lengths involving heavy lanthanum atoms. The L2 sample exhibits a higher shoulder at 3.80 Å and a lower peak at 4.20 Å with respect to the L1 sample.

**Figure 5.**
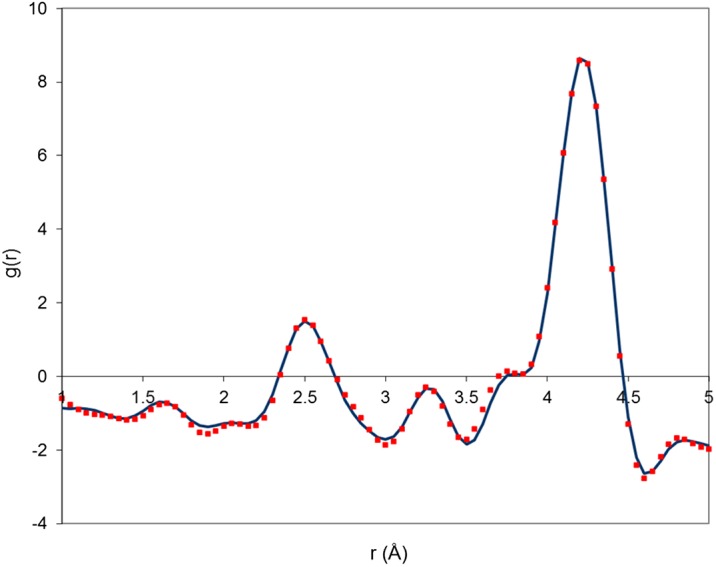
Experimental PDF of sample L1 (squares) and PDF calculated from model M1 (solid line)**.**

The experimental PDF of the sample L1 is compared to that calculated from model M1 in Figure 5. The agreement between the two curves is very good, especially since no refinement was performed (only three isotropic displacement parameters were adjusted). The main discrepancy is related to the shoulder at 3.80 Å, which appears at too large r values for the model with respect to experiment. However, this difference is small, and it should be remembered that the L1 sample contains a small amount of excess oxygen. Overall, these results confirm that DFT is able to produce good-quality structure models independently of experimental data and that our models capture most of the interatomic distances present in the real lanthanum silicate structure.

**Figure 6.**
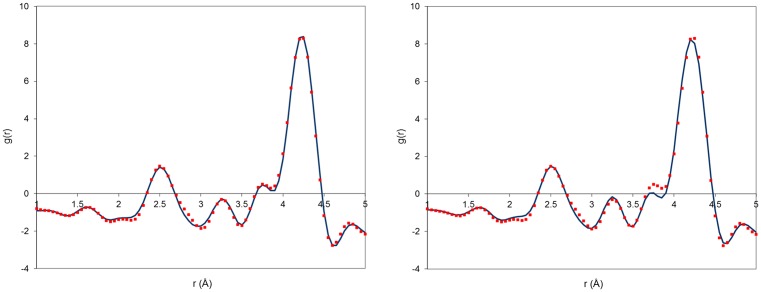
Experimental PDF of sample L2 (squares) compared to PDF calculated (solid line) from model M2 (left image) and model M3 (right image)**.**

**Figure 7.**
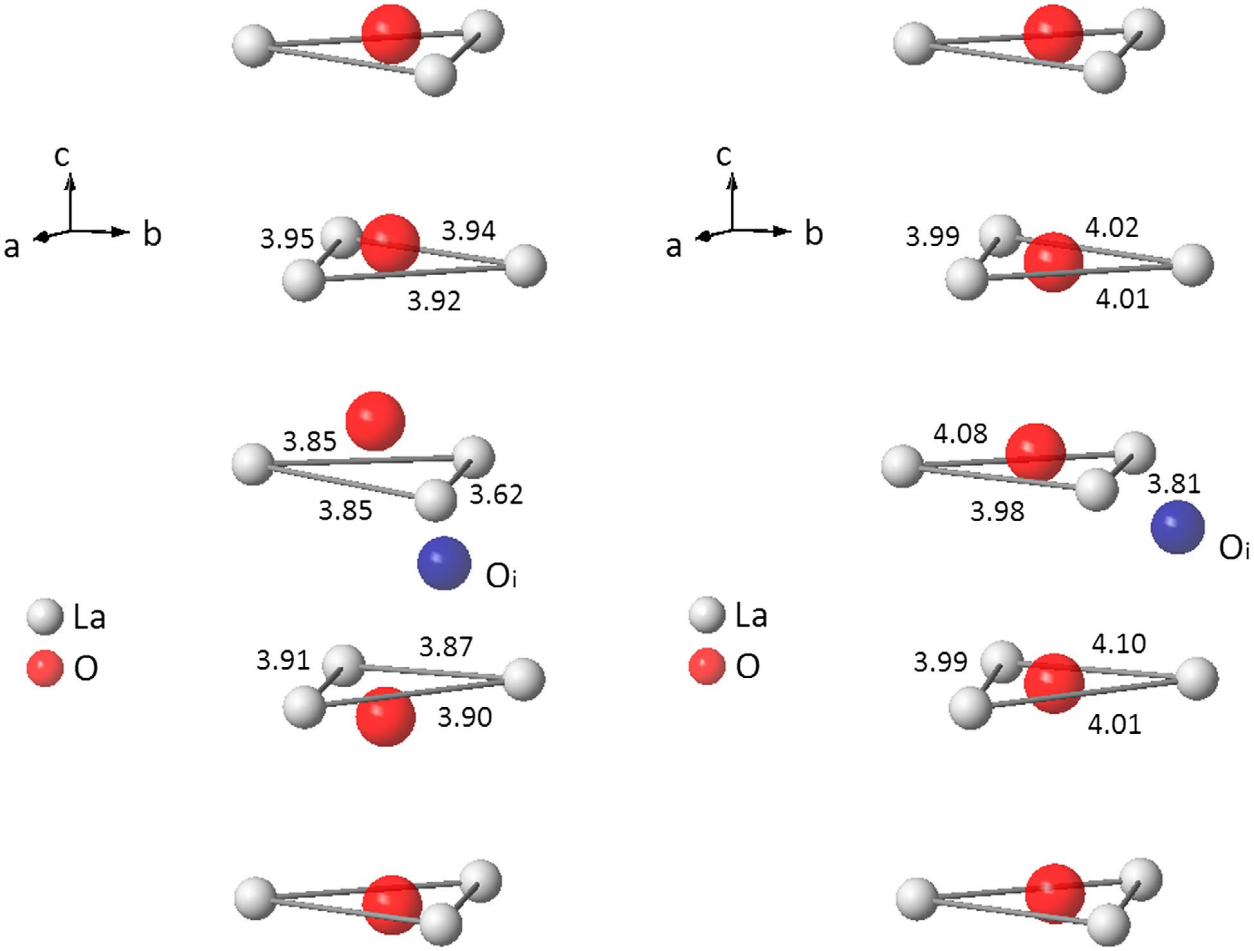
Perspective views of the conduction channel, showing the O4 oxide ions, the La2 lanthanum triangles, the Oi interstitial oxide ion and some triangle edge lengths for model M2 (left image) and model M3 (right image)**.**

The experimental PDF of the sample L2 is now compared to the PDF calculated from the model M2 and M3 in Figure 6. Differences are clearly visible between the two models. The model M2 is able to reproduce accurately both the heights and positions of the shoulder at 3.80 Å and the peak at 4.20 Å. This is not the case with the model M3, which produces too low a shoulder at 3.80 Å and slightly too broad a peak at 4.20 Å. The determination of the exact change in La-La correlations responsible for the correct evolution from samples L1 to L2 requires a close inspection of the structure models around the conduction channel, in particular the L2 triangle surrounding the O4 sites. The perspective views of the conduction channel, showing the O4 oxide ions, the La2 lanthanum triangles, the Oi interstitial oxide ion and some triangle edge lengths for the models M2 and M3 are plotted in Figure 7.

Again, differences between the models M2 and M3 are clearly visible. In the model M2, the interstitial oxide ion pushes nearby O4 ions far away from the La2 triangle centres. This not only induces the large U_33_ value discussed in the previous section but also induces a contraction of the La2 triangles and a large shortening of the La2-La2 edges. The calculated average La2 triangle area is 6.74 Å^2^. It compares very well with that measured for sample L2 (6.78 Å^2^). In addition, over the 24 triangle edges of the M2 supercell, 4 edges have length smaller than 3.90 Å, resulting in about 17% of edges with an average length of 3.80 Å instead of 4.02 Å as found in model M1. Thus, when inserting excess oxide ions within the channel (from M1 to M2), 17% of the La-La distances that were contributing to the left side of the peak at 4.20 Å in model M1 contribute henceforth in model M2 to the shoulder at 3.80 Å. This explains the higher shoulder at 3.80 Å and a lower peak at 4.20 Å observed for the PDF of the model M2 and sample L2 with respect to the model M1 and sample L1. The model M3 is clearly unable to explain these observations. Only small changes are observed in the conduction channel. The shortening of a single triangle edge is partly compensated by the lengthening of another, so that the calculated average triangle area is 7.02 Å^2^, significantly larger than that measured for sample L2. For the sake of completeness, it should be pointed out that the increase of the number of La1 atoms, going from compositions La_9.33_(SiO_4_)_6_O_2_ to La_9.50_(SiO_4_)_6_O_2.25_, induces a small decrease of the average La1-La1 closest distances, which goes from 3.73 Å in the model M1 to 3.69 Å in the models M2 and M3. This explains the small shift of the shoulder at 3.80 Å towards lower r values.

The results presented so far all indicate that the structural changes induced by incorporating excess oxygen in the lanthanum silicate structure are consistent with interstitial oxide ions located within the conduction channel and not at its periphery. This, in turn, is consistent with the interstitialcy diffusion mechanism along the c-axis involving cooperative movement of O4 and Oi ions [12]. It should also be kept in mind that the present results are based on calculations or experiments corresponding to relatively low temperatures (0 K for calculations and mostly 15 K and room temperature for experimental data) with respect to the operating temperatures of SOFC. Thus, it is not excluded that other conduction mechanisms may also occur in operating conditions, in particular those favouring the formation of SiO_5_ units or even more complex ones such as the one reported recently in literature [15].

## 5. Conclusions

We have investigated the local structure of apatite-type lanthanum silicates by combining the PDF method, conventional X-ray and neutron diffraction data and DFT calculations. It was shown that DFT is able to produce, independently of experimental data, good-quality structure models which capture the disorder present in the lanthanum silicate compounds. It was also shown that both the average and local structures of the La_9.50_(SiO_4_)_6_O_2.25_ compound are best represented by the model M2, with Oi within the conduction channel and not at its periphery. With this model, the experimental ADP values of the O3 and O4 ions are well explained. The high observed value of U_33_ of O4 appears to be the key signature of interstitial oxide ions within the channel. In addition, the large observed U_11_ value of O3 is shown not to be the signature of an interstitial ion located at the periphery of the channel as reported in literature. An interstitial ion located at the channel periphery would indeed induce a much larger increase of O3 ADP values than experimentally observed. It also appears that the interstitial oxide ions within the channel not only induce the large U_33_ value of O4 but also a contraction of the La2 triangles and a large shortening of the La2-La2 edges. This explains perfectly the La2 triangle area measured experimentally and the height of the experimental PDF shoulder at 3.80 Å.

Thus, the obtained results all indicate that the structural changes measured by diffraction methods and reported in literature or in the present study are consistent with interstitial oxide ions located within the conduction channel and not at its periphery. This, in turn, is consistent with a conduction mechanism along the c-axis involving cooperative movement of O4 and Oi ions. As already stated, it should also be kept in mind that the present results are based on calculations or experiments corresponding to relatively low temperatures (0 K for calculations and mostly 15 K and room temperature for experimental data) with respect to the operating temperatures of SOFC. Thus, it is not excluded that other conduction mechanisms may also occur in operating conditions.

## Disclosure statement

No potential conflict of interest was reported by the authors.

## Supplemental data

The supplemental material for this paper is available online at https://doi.org/10.1080/14686996.2017.1362939

## Supplementary Material

supporting_information.docx
