# Supplementary material for: Local structure and oxide-ion conduction mechanism in apatite-type lanthanum silicates
Source: Sci Technol Adv Mater. 2017 Sep 4;18(1):644–53. doi: 10.1080/14686996.2017.1362939 (PMC5614213; doi:10.1080/14686996.2017.1362939)
Supplement: supporting_information.docx [file TSTA_A_1362939_SM7297.docx]

**Supporting information**


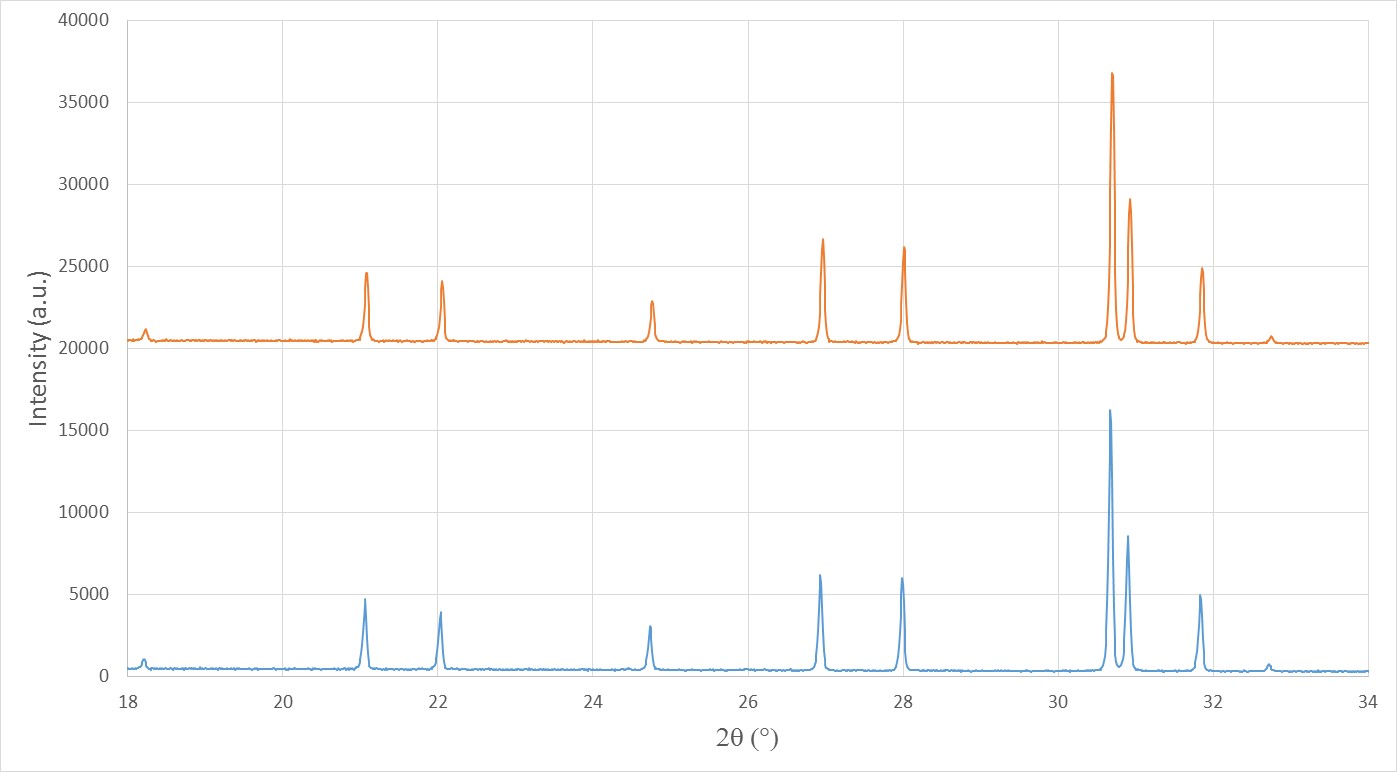


**Fig. S1.** Low angle diffraction patterns of the samples L1 (lower curve) and L2 (upper curve). No trace of secondary phases are visible.


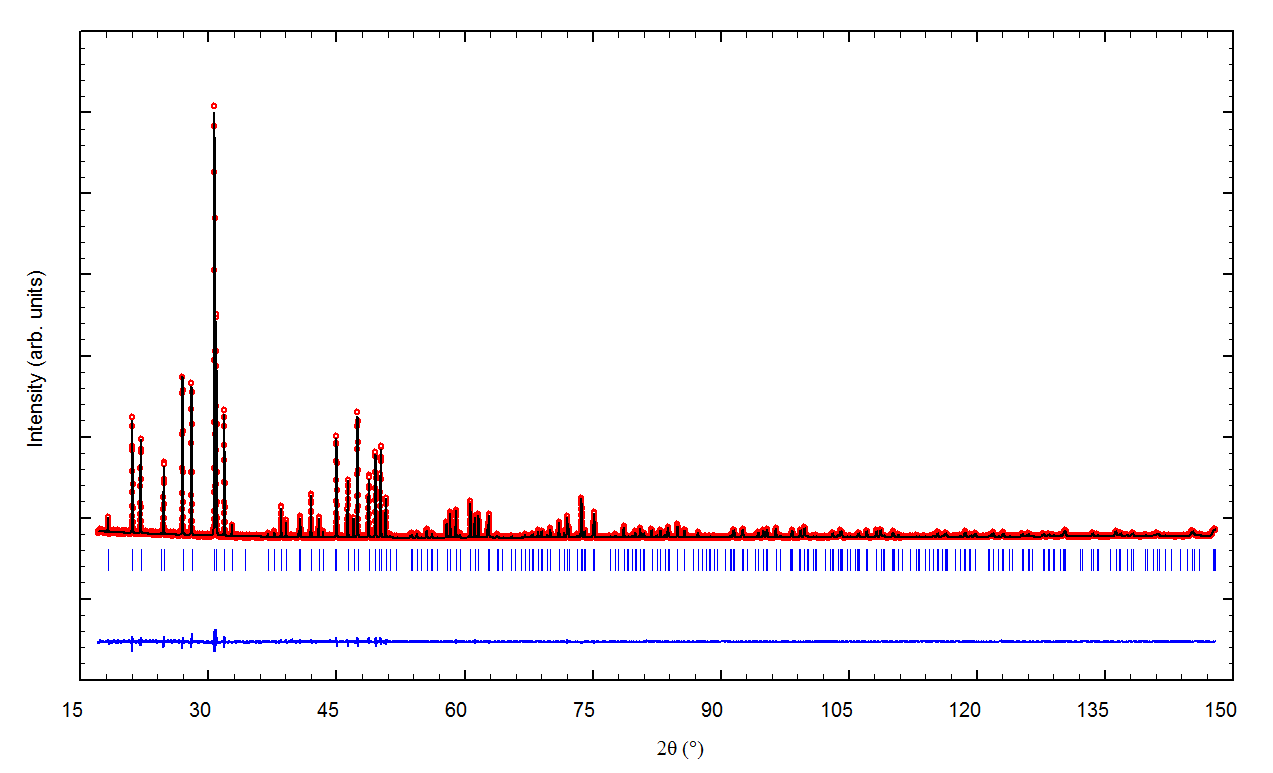


**Fig. S2.** Rietveld fit for the L1 sample: observed (open circle), calculated (solid line) and difference patterns
